# Perceived neighborhood illicit drug selling, peer illicit drug disapproval and illicit drug use among U.S. high school seniors

**DOI:** 10.1186/1747-597X-9-35

**Published:** 2014-09-03

**Authors:** Dustin T Duncan, Joseph J Palamar, James H Williams

**Affiliations:** 1 Department of Population Health, New York University School of Medicine, 227 East 30th Street, 6th Floor, Room 621, New York, NY 10016, USA; 2 Center for Drug Use and HIV Research, New York University College of Nursing, New York, NY, USA; 3 Population Center, New York University, New York, NY, USA; 4 Global Institute of Public Health, New York University, New York, NY, USA

**Keywords:** Neighborhood drug selling, Peer drug attitudes, Illicit drug use, Adolescents

## Abstract

**Background:**

This study examined associations between perceived neighborhood illicit drug selling, peer illicit drug disapproval and illicit drug use among a large nationally representative sample of U.S. high school seniors.

**Methods:**

Data come from Monitoring the Future (2007–2011), an annual cross-sectional survey of U.S. high school seniors. Students reported neighborhood illicit drug selling, friend drug disapproval towards marijuana and cocaine use, and past 12-month and past 30-day illicit drug use (*N* = 10,050). Multinomial logistic regression models were fit to explain use of 1) just marijuana, 2) one illicit drug other than marijuana, and 3) more than one illicit drug other than marijuana, compared to “no use”.

**Results:**

Report of neighborhood illicit drug selling was associated with lower friend disapproval of marijuana and cocaine; e.g., those who reported seeing neighborhood sales “almost every day” were less likely to report their friends strongly disapproved of marijuana (adjusted odds ratio [AOR] = 0.38, 95% CI: 0.29, 0.49) compared to those who reported never seeing neighborhood drug selling and reported no disapproval. Perception of neighborhood illicit drug selling was also associated with past-year drug use and past-month drug use; e.g., those who reported seeing neighborhood sales “almost every day” were more likely to report 30-day use of more than one illicit drug (AOR = 11.11, 95% CI: 7.47, 16.52) compared to those who reported never seeing neighborhood drug selling and reported no 30-day use of illicit drugs.

**Conclusions:**

Perceived neighborhood drug selling was associated with lower peer disapproval and more illicit drug use among a population-based nationally representative sample of U.S. high school seniors. Policy interventions to reduce “open” (visible) neighborhood drug selling (e.g., problem-oriented policing and modifications to the physical environment such as installing and monitoring surveillance cameras) may reduce illicit drug use and peer disapproval of illicit drugs.

## Background

National estimates show that approximately 45.5% of U.S. high school seniors report using marijuana one or more times during their life, over a third (36.4%) report using in the past 12 months, and almost one-quarter (22.7%) report using marijuana one or more times during the past 30 days [1]. Use of other illicit drugs is less prevalent. For example, 4.5% of U.S. high school seniors report ever using cocaine one or more times during their life, 2.6% report using in the past 12 months, and 1.1% report using cocaine one or more times during the past 30 days [1]. Drug use in adolescence (especially late adolescence) is associated with increased risk for drug use disorders as well as other health and social problems such as school failure and sexually transmitted infections including HIV [2-4].

For decades, drug use research has focused largely on individual (e.g., gender), family (e.g., family support) and peer (e.g., peer groups) factors in explaining illicit drug use; however, unexplained variance exists [5]. A relatively under-explored aspect of risk factors for illicit drug use is one’s neighborhood. Social epidemiology research shows that neighborhood environments can play a significant role in drug use [5,6]. For example, previous studies have examined associations between neighborhood socio-demographic characteristics (e.g., neighborhood poverty) and illicit drug use [7-14]. In addition, prior research shows that exposure to neighborhood violence and crime is associated with illicit drug use [15-17]. Using and analyzing aggregate measures of neighborhood social disorder (including neighborhood drug selling as a component), some other studies have found a relationship between neighborhood social disorder and illicit drug use behaviors [16,18-20]. A recent study by Epstein and colleagues (2014) showed that neighborhood-level drug activity and social disorder were associated with heroin and cocaine craving among drug misusers [21].

The use of aggregate measures of neighborhood disorder is described in the sociology and public health literature including studies from the Project on Human Development in Chicago Neighborhoods (PHDCN) [22-26]. Multidimensional measures of social disorder are based on Raudenbush’s ecometric theory, i.e., the reliability of a scale improves as you observe multiple indicators, as opposed to single indicators [27]. In addition, the indicators are often clustered with each other and tend to co-occur. PHDCN measures of social disorder (based on ecometrics) are applied to indicators obtained from systematic social observation. However, when measuring a single individual’s evaluation of neighborhood characteristics (as opposed to using systematic social observation) it is may not be necessary to apply aggregate measures of neighborhood characteristics (e.g. neighborhood disorder) [28]. In addition to neighborhood drug selling, aggregate measures of neighborhood social disorder often include the following other components: individuals drinking alcohol in public, individuals using or being addicted to drugs, unemployed individuals hanging out in the streets, and prostitutes on the street [22,29].

While informative, the previous research on the role of neighborhood factors and illicit drug use has typically not explicitly and specifically examined neighborhood illicit drug selling, a potentially important factor in illicit drug use on its own. Neighborhood illicit drug selling is a distinctive indicator of neighborhood social disorder that may be amenable to policy. We recognize that composite measures (e.g., of neighborhood social disorder) can be important, but might diminish the importance of any one individual component and might be less transferable to policymakers [30-32] (as studies usually use and analyze aggregate measures of neighborhood social disorder—not stratifying by the myriad social disorder components such as neighborhood drug selling) [16,18-20]. Further, while previous research on neighborhood factors and illicit drug use has used a variety of methods to categorize neighborhood factors (e.g., survey, geographic information systems [GIS]), additional research is needed to examine perceptions of neighborhood characteristics, which may be more closely linked to health and behavior than objectively measured neighborhood characteristics. It is also important to note that, based on our review of the accumulated literature, most previous research on neighborhood factors in illicit drug use has used non-representative local populations, which limits generalizability. We also note that neighborhood factors might not only influence illicit drug use, but drug-related attitudes. Previous studies have examined demographic (e.g., age) and behavioral (e.g., drug use) correlates of peer drug-related attitudes such as peer illicit drug disapproval [33,34], however, to our knowledge, studies have not examined the effect of neighborhood characteristics on peer drug-related attitudes and thus represents a critical gap in the literature. Importantly, sociological and psychological theories, such as Social Norms Theory [35] and the Theory of Planned Behavior [36], suggest that neighborhood illicit drug selling could be associated with peer illicit drug disapproval. In addition, disapproval and stigmatization towards illicit drug use has previously been shown to be an important correlate of illicit drug use [37]. Research on how perceived neighborhood drug selling relates to both use and (peer) attitudes towards use would add to the literature as there is a lack of information whether perception of neighborhood drug selling is a risk factor for use. Potential mechanisms linking neighborhood drug selling and drug use could be availability of drugs, perceived normality of use, and perhaps pressure from drug dealers to use drugs. In addition, neighborhood drug selling and concomitant issues (including neighborhood violence) could be stressful and therefore influence drug use. For example, in the context of neighborhood stress, adolescents might use marijuana given its well-established anxiolytic effects.

The primary aim of this study is to examine the association of perceived neighborhood illicit drug selling and illicit drug use among a large population-based nationally representative sample of U.S. high school seniors. Because peers (e.g., proportion of school friends using illicit drugs) can influence illicit drug use among adolescents [38,39], we additionally evaluated the association of perceived neighborhood illicit drug selling and peer illicit drug disapproval among the sample. Based on previous theoretical and empirical research, we hypothesized that perceived neighborhood illicit drug selling would be associated with higher odds for illicit drug use and lower peer illicit drug disapproval among our sample of U.S. high school seniors.

## Methods

### Data

Monitoring the Future (MTF) is an annual cross-sectional survey of high school seniors in approximately 130 public and private schools throughout 48 states in the US [40]. Schools are selected through a multi-stage random sampling procedure: geographic areas are selected, then schools within areas are selected, and finally students within schools are selected. MTF assesses content using six different survey forms, which are distributed randomly. All forms assess demographic characteristics and drug use, however, only survey Form 4 assesses perception of drug selling in one’s neighborhood and friend disapproval towards use of various drugs. To increase power, we combined data from the most recent five cohorts (2007–2011) of data into a single cross-section--consistent with many previous MTF studies [34,41-48]. MTF protocols were reviewed and approved by the University of Michigan Institutional Review Board (IRB). We received IRB approval to examine data on MTF seniors from New York University School of Medicine.

### Neighborhood illicit drug selling

Students were asked, “During the past 12 months, how often have you seen people selling illegal drugs in your neighborhood?” Answer options were: 1) “Never”, 2) “A few times a year”, 3) “Once or twice a month”, 4) “At least once a week”, and 5) “Almost every day”. Answers were coded into indicator variables with “never” as the comparison.

### Peer illicit drug disapproval

Students were asked about their perception of friend disapproval towards trying various illicit drugs, including marijuana and cocaine. To assess disapproval towards marijuana, students were asked: “How do you think your close friends feel (or would feel) about you trying marijuana (pot, weed) once or twice?” Answer options were: 1) “Don’t disapprove,” 2) “Disapprove” and 3) “Strongly disapprove.” A similar question was asked with regard to use of cocaine. These two variables were coded into indicators: “disapprove” and “strongly disapprove” and “don’t disapprove” served as the comparison.

### Past 12-month and past 30-day illicit drug use

MTF asked students about use of various illicit drugs that occurred within the past 12 months and within the past 30 days. Students were asked whether they used marijuana (pot, weed, hashish), and other illicit substances including cocaine, crack, LSD, hallucinogens other than LSD, heroin, MDMA (ecstasy, “Molly”) and nonmedical use of narcotics (other than heroin), tranquilizers (e.g., benzodiazepines), sedatives (e.g., barbiturates) and amphetamine. We then created a new variable with four categories (one for 12 month use and one for 30 day use): 1) no illicit drug use, 2) only marijuana use, 3) use of one illicit drug other than marijuana, and 4) use of more than one illicit drug other than marijuana. We combined use of multiple drugs into one category as an indicator of drug use severity.

### Covariates

Students indicated their age (dichotomized by MTF as <18 and ≥18 years), sex (male vs. female), and race/ethnicity (defined by MTF as Black, White and Hispanic). MTF classified population density of students’ residences as non-, small-, or large-metropolitan statistical areas (MSAs). Small MSAs are defined as counties or groups of counties with at least one city of 50,000 or more inhabitants and the 24 largest MSAs are defined as large MSAs [40]. The remaining areas are defined as non-MSAs. MTF also assessed level of religious attendance and importance through two ordinal items. We created a mean composite of these two items (range: 1–4) and divided the scores into tertiles representing low (1.0-2.0), moderate (2.5-3.0) and high (3.5-4.0) religiosity. Students were also asked about level of educational attainment of each parent through an ordinal item. We computed a mean score for both parents (or raw score if only one parent) and this was further coded into three groups representing low (1.0-3.0), medium (3.5-4.0), and high (4.5-6.0) education. Students were also asked to indicate the number of evenings they usually go out per week for fun and recreation. We recoded their responses to the ordinal item into: 1) 0–1 evening(s), 2) 2–3 evenings, and 3) 4–7 evenings. These covariates were identified a priori and coding of covariates was based on previous MTF analyses [43,49-52].

### Statistical analyses

Analyses focused on MTF senior students with complete neighborhood illicit drug selling and peer disapproval data (Unweighted *N* = 10,050; Weighted *N* = 10,089). Some statistically significant differences between participants with completed neighborhood illicit drug selling and peer disapproval data as compared with the entire sample existed. However, given the large size of the dataset this is not surprising. Because missing data can be common and problematic, we evaluated missingness in the covariates. Since some covariates were missing data (e.g., race/ethnicity [missing 14.3%], religiosity [missing 23.0%]), we allowed for covariates to have missing data in the regression models. In Table 1, we have included the number of missing values for each covariate: The range of missing data for our covariates was 0.3% (age) to 23% (religiosity). These data were missing not at random. We entered missing data indicators to ensure that these cases were not deleted. This method has been used in previous MTF analyses [42,51-53] to maintain power and representativeness of the sample. Only 47.4% of the analytic sample had case-compete data; therefore, deleting cases with any missing data would have resulted in the listwise deletion of more than half the sample, reducing power and it’s national representativeness.

**Table 1:** **Sample characteristics (unweighted *N*** **= 10,050)**

**Variable**	***N***	**Unweighted %**
Age, years		
< 18 years	4,340	43.2
≥ 18 years	5,678	56.5
Missing	32	0.3
Sex		
Male	4,521	44.9
Female	5,129	51.3
Missing	400	3.8
Race/ethnicity		
White	6,114	62.3
Black	1,092	10.6
Hispanic	1,403	13.8
Missing	1,441	13.4
Population density		
Non-Metropolitan Statistical Area (MSA)	2,007	22.4
Small MSA	4,673	49.2
Large MSA	3,370	28.4
Religiosity		
Low	3,124	31.2
Moderate	2,228	21.5
High	2,386	24.4
Missing	2,312	23.0
Parent education		
Low	2,870	28.7
Moderate	2,853	28.9
High	4,027	39.4
Missing	300	3.0
Evenings out per week for fun		
0-1	2,652	27.3
2-3	5,127	50.5
4-7	2,188	21.3
Missing data	83	0.9
Friend marijuana disapproval		
None	4,295	41.5
Disapproval, but not strongly	2,325	23.0
Strongly disapprove	3,430	35.5
Friend cocaine disapproval		
None	994	9.8
Disapproval, but not strongly	1,843	18.1
Strongly disapprove	7,213	72.1
Perceived neighborhood drug sales		
Never	6,120	61.8
A few times per year	1,607	15.7
Once or twice a month	769	7.7
One or more per week	779	7.3
Almost every day	775	7.6
12-month illicit drug use		
None	6,372	64.0
Use of marijuana only	1,972	18.8
Use of one illicit drug	747	7.3
Use of more than one illicit drug	959	9.9
30-day illicit drug use		
None	7,803	77.8
Use of marijuana only	1,412	13.8
Use of one illicit drug	440	4.4
Use of more than one illicit drug	395	4.1

We examined descriptive statistics (e.g., weighted percentages) for each covariate and then we fit all variables into multinomial multivariable logistic regression models with the four-category drug use variable as the outcome (one model for 12 month use and another model for 30 day use). The comparison variable for both outcome variables was “no use.” These models determined the conditional associations of perceived neighborhood drug selling and peer drug disapproval while controlling for all other covariates. The predictors in the model explain use of 1) just marijuana, 2) one illicit drug other than marijuana, and 3) more than one illicit drug other than marijuana, compared to “no use,” similar to multiple binary logistic regressions. This way each predictor is associated with an adjusted odds ratio (AOR) and 95% confidence interval (CI) for each of the three levels of the outcome variable. We present AORs and CIs for perceived neighborhood drug selling and peer disapproval. Data indicators for cohort (with year 2007 as the comparison) were entered into all models to control for potential cohort effects and/or secular trends. Data were weighted to adjust for differential probability of selection of schools and students. All analyses were design-based for survey data using Taylor series variance estimate (PROC SURVEYLOGISTIC) [54] and conducted using SAS 9.3 software (SAS Institute Inc., Cary, NC).

## Results

Table 1 shows the characteristics of the sample of 10,050 U.S. high school seniors. More than half (56.5%) of the sample was ≥18 years old. About half (51.3%) were female and most (62.3%) were White. Almost 62% of students reporting “never” seeing neighborhood drug sales, and 15.7% reporting seeing neighborhood drug sales “a few times per year”. Almost 60% of the sample reported that their friends disapprove of marijuana use, of which 35.5% reported that their friends “strongly disapprove”. On the other hand, 90.2% of the sample reported that their friends disapprove of cocaine use; 72.1% of the sample reported that their friends “strongly disapprove” of cocaine.

The majority of the sample reported no past-year illicit drug use and past-month illicit drug use: 9.9% reported use of more than one illicit drug in the past 12 months, and 4.1% reported use of more than one illicit drug in the past month. The majority of users of illicit drugs other than marijuana also reported used of marijuana (data not presented in table). Specifically, for both 12-month and 30-day users of one illicit drug other than marijuana, all (100%) also reported 12-month/30-day use of marijuana. With regard to use of more than one illicit drug, 66.5% of 12-month users also reported 12-month use of marijuana, and with respect to 30-day use of more than one other illicit drug, 58.3% also reported 30-day use of marijuana.

### Neighborhood illicit drug selling and peer illicit drug disapproval

Report of neighborhood illicit drug selling was associated with lower friend disapproval of marijuana and cocaine in multivariable models, controlling for socio-demographic factors/ other covariates (Tables 2 and 3). For example, those who reported seeing neighborhood sales “almost every day” were at lower odds of reporting that friends disapproved (AOR = 0.36, 95% CI: 0.28, 0.47) and strongly disapproved (AOR = 0.38, 95% CI: 0.29, 0.49) of marijuana compared to those who reported never seeing neighborhood drug selling and reported no disapproval (Table 2). Similarly, neighborhood illicit drug selling was associated with lower friend disapproval of cocaine (Table 3). Those who reported seeing neighborhood sales “almost every day” were at lower odds of reporting that their friends disapproved (AOR = 0.57, 95% CI: 0.44, 0.77) and strongly disapproved (AOR = 0.40, 95% CI: 0.31, 0.52) of cocaine compared to those who reported never seeing neighborhood drug selling and reported no disapproval, for example. In addition, as shown in Tables 2 and 3, odds for strong friend disapproval towards use of each drug tended to be more robust (smaller) than non-strong friend disapproval (“disapprove”), when significant. Perceiving drug selling once or more per week (or almost every day) was significantly (negatively) associated with friend cocaine disapproval, but almost all levels of perceived selling in one’s neighborhood were significantly negatively associated with friend marijuana disapproval.

**Table 2:** Association between neighborhood illicit drug selling and friend disapproval towards trying marijuana

	**Disapprove (N = 2,325)**		**Strongly disapprove (N = 3,430)**	
	**AOR**	** (95% CI)**	**AOR**	** (95% CI)**
Perceived neighborhood drug sales				
Never	1.00		1.00	
A few times per year	0.97	(0.82, 1.15)	0.74***	(0.63, 0.88)
Once or twice a month	0.54***	(0.43, 0.68)	0.49***	(0.38, 0.62)
One or more per week	0.57***	(0.45, 0.72)	0.45***	(0.36, 0.58)
Almost every day	0.36***	(0.28, 0.47)	0.38***	(0.29, 0.49)

Note: Comparison is no disapproval, N = 4,295.

*= p<.05, **= p<.01, ***= p<.001.

**Table 3:** Association between neighborhood illicit drug selling and friend disapproval towards trying cocaine

	**Disapprove (N = 1,843)**		**Strongly disapprove (N = 7,213)**	
	**AOR**	** (95% CI)**	**AOR**	** (95% CI)**
Perceived neighborhood drug sales				
Never	1.00		1.00	
A few times per year	0.90	(0.68, 1.18)	0.90	(0.71, 1.14)
Once or twice a month	0.96	(0.69, 1.36)	0.89	(0.66, 1.20)
One or more per week	0.58***	(0.42, 0.79)	0.58***	(0.44, 0.75)
Almost every day	0.57***	(0.44, 0.77)	0.40***	(0.31, 0.52)

Note: Comparison is no disapproval, N = 994.

*= p<.05, **= p<.01, ***= p<.001.

### Neighborhood illicit drug selling and illicit drug use

The multivariable associations between neighborhood illicit drug selling and past-year drug use are reported in Table 4, controlling for socio-demographic factors/other covariates and peer disapproval of illicit drugs. Those who reported seeing neighborhood sales “almost every day” were more likely to report 12-month use of marijuana (AOR = 2.31, 95% CI: 1.74, 3.09), 12-month use of one illicit drug (AOR = 2.70, 95% CI: 1.88, 3.89), and 12-month use of more than one illicit drug (AOR = 6.19, 95% CI: 4.43, 8.64) compared to those who reported never seeing neighborhood drug selling and reported no 12-month use of illicit drugs. Those who reported seeing neighborhood sales “almost every day” were more likely to report 30-day use of marijuana (AOR = 3.58, 95% CI: 2.74, 4.67), 30-day use of one illicit drug (AOR = 3.91, 95% CI: 2.69, 5.70), and 30-day use of more than one illicit drug (AOR = 11.11, 95% CI: 7.47, 16.52) compared to those who reported never seeing neighborhood drug selling (Table 5). In addition, as shown in Tables 4 and 5, the effects of reporting neighborhood selling of illicit drugs was stronger for past 30-day use of illicit drugs and strongest for those who used more drugs. With regard to 12-month use, all levels of perceived neighborhood drug selling tended to be associated with higher odds of only marijuana use than use of only one other illicit drug; however, odds tended to be much higher for use of multiple other illicit drugs (Table 4). With respect to 30-day use, the more frequent the witnessing of neighborhood drug selling, the higher the odds for drug use (Table 5). Odds also increased accordingly for use of more illicit drugs other than marijuana (e.g., frequent exposure to selling was associated with increased odds of only marijuana use, even higher odds for other illicit drug use, and much higher odds for use of multiple other illicit drugs in the past 30 days) (Tables 4 and 5).

**Table 4:** Association between neighborhood illicit drug selling and illicit drug use in past 12 months

	**12-Month use of marijuana (No other illicit drug use) (N = 1,972)**		**12-month use of one illicit drug (N = 747)**		**12-month use of 2–10 illicit drugs (N = 959)**	
	**AOR**	**(95% CI)**	**AOR**	**(95% CI)**	**AOR**	**(95% CI)**
Perceived neighborhood drug sales						
Never	1.00		1.00		1.00	
A few times per year	1.31*	(1.07, 1.61)	1.43**	(1.10, 1.85)	1.34	(1.00, 1.80)
Once or twice a month	2.43***	(1.86, 3.17)	2.41***	(1.73, 3.35)	3.49***	(2.52, 4.82)
One or more per week	3.27***	(2.53, 4.23)	3.20***	(2.30, 4.45)	6.44***	(4.63, 8.96)
Almost every day	2.31***	(1.74, 3.09)	2.70***	(1.88, 3.89)	6.19***	(4.43, 8.64)

Note: Comparison is 12-month use of no illicit drugs, N = 6,372.

*= p<.05, **= p<.01, ***= p<.001.

**Table 5:** Association between neighborhood illicit drug selling and no illicit drug use in past 30 days

	**30 day use of marijuana (no other illicit drug use) (N = 1,412)**		**30 day use of one illicit drug (N = 440)**		**30 day use of 2–10 illicit drugs (N = 395)**	
	**AOR**	**(95% CI)**	**AOR**	**(95% CI)**	**AOR**	**(95% CI)**
Perceived neighborhood drug sales						
Never	1.00		1.00		1.00	
A few times per year	1.30*	(1.03, 1.64)	1.43*	(1.03, 1.98)	1.64*	(1.03, 2.63)
Once or twice a month	2.02***	(1.58, 2.58)	1.68**	(1.15, 2.45)	2.79***	(1.82, 4.25)
One or more per week	2.84***	(2.20, 3.67)	3.21***	(2.18, 4.71)	5.17***	(3.44, 7.78)
Almost every day	3.58***	(2.74, 4.67)	3.91***	(2.69, 5.70)	11.11***	(7.47, 16.52)

Note: Comparison is 30 day use of no illicit drugs, N = 7,803.

*= p<.05, **= p<.01, ***= p<.001.

## Discussion

Adolescence is an important developmental period for the initiation of illicit drugs [4,55,56]. In this study, among a population-based nationally representative sample of U.S. high school seniors, we found that perception of neighborhood illicit drug selling was associated both with illicit drug use and peer disapproval of illicit drugs. Specifically, report of neighborhood illicit drug selling was associated with lower peer disapproval and more illicit drug use. Furthermore, increased frequency of witnessing neighborhood drug-dealing is associated with “more severe” use (e.g., more recent use, use of drugs “harder” than marijuana and multiple illicit drugs). In addition, we found that increased frequency of witnessing drug selling was associated with lower levels of friend disapproval toward use of marijuana and cocaine. Disapproval (self-, peer-, and perceived societal disapproval) has previously been found to be a robust protective factor against drug use [34,37,57-60]. These findings suggest that increased frequency of witnessing drug selling in one’s neighborhood is associated with lowered friend disapproval toward marijuana, but much higher frequency of witnessing drug selling was needed in order for students to report significantly lower friend disapproval toward cocaine. Marijuana is the least stigmatized or disapproved illicit drug and cocaine use is more heavily stigmatized [37]. For example, 42.1% of young adults (age 23–26) disapprove of an adult trying marijuana, but 86.0% of young adults disapprove of an adult trying cocaine [61]. While use of a drug is generally associated with decreased disapproval or stigma toward use [34,37], these findings add to our understanding in that witnessing drug selling in one’s neighborhood is also associated with decreases in friend drug disapproval.

Our study adds to the burgeoning literature on the role of the neighborhood social context on illicit drug use among adolescents. By and large, our findings are consistent with existing studies evaluating the role of neighborhood environments in illicit drug use [16,18-20]. However, because aggregate measures of neighborhood social disorder include other factors such as alcohol use, drug use, drug addiction, and prostitution, it is unclear whether multiple aspects of neighborhood social disorder are simultaneously needed to detect significant effects on illicit drug use. While multiple aspects of social disorder may contribute to illicit drug use, aggregate measures obscure the importance of any one particular aspect on illicit drug use. To our knowledge, this is the first study to examine the effect of a neighborhood characteristic on peer drug attitudes and thus our study provides a new contribution to the literature.

There are a variety of potential explanations for our study findings. First, neighborhood characteristics such as neighborhood drug selling may influence *social norms*, which, in turn, may influence perception of drug use and abuse and ones use and abuse of drugs [37]. Indeed, it is possible that individuals begin to become desensitized to drug use when they frequently witness drug sales. When drug use or drug selling becomes somewhat of a normalized activity in one’s view it thus seems that disapproval towards use decreases, leaving individuals at higher risk for drug use. Second, neighborhood drug selling and concomitant issues (including neighborhood violence) might be also *stressful* and therefore influence drug use [7,16,62,63]. Third, witnessing drug selling may also be an indicator of *drug availability* in one’s neighborhood, potentially leading to use and abuse of drugs. For instance, exposure to users and familiarity with users has previously been found to be robust risk factors for drug use [60,64]. Furthermore, neighborhood drug dealers might *pressure* purchase of drugs and drug use among adolescents as well as other community members. Students reporting neighborhood drug selling may be more likely to know the drug dealers, notice selling, or even purchase or sell drugs themselves. In addition to confirming findings from our study in other samples, future research should identify mechanisms through which neighborhood drug selling might contribute to increases in illicit drug use among adolescents, including the influence of neighborhood drug selling on social norms. Future studies should also examine temporal associations, which are needed to more carefully examine the direction of association.

Findings from this study may be relevant to practice and policy, including the potential need for neighborhood-level policy changes. Empirical evidence from the criminology and public health literature shows that drug-related crime can be decreased by modifying the social and physical neighborhood environment through a variety of strategies [65-68]. Policies monitoring illicit drugs may reduce the availability of illicit drugs and therefore reduce neighborhood drug selling. For example, local police attention in “hot spots” or areas common for neighborhood drug selling (known as problem-oriented policing) may be a useful strategy for reducing “open” (visible) neighborhood drug selling [65,66,68]. In addition, adjustments to the physical environment (e.g., installing and monitoring surveillance cameras and landscaping trees and shrubs) might also prove to be beneficial in reducing open neighborhood drug selling [67,68]. It should be noted that even in states where marijuana use is decriminalized, sales and use in “public view” are still illegal [69]. Our study demonstrates the role of neighborhood factors in shaping drug use among adolescents, and past evidence showing the effectiveness neighborhood-oriented policing approaches suggests that neighborhood-level policy changes may help reduce illicit drug use among this population.

### Limitations

First, we recognize that reverse causality is a possibility: individuals who used illicit drugs may report higher rates of neighborhood drug sales. As these are cross-sectional data, temporal ambiguity is a concern; longitudinal study designs are needed to provide evidence of temporal ordering. Additionally, natural experiments or policy changes (e.g., police efforts targeting neighborhood drug selling) could be evaluated and would provide the strongest evidence for causality. As previously discussed, neighborhood-level factors can be measured in a variety of ways, including objectively via systematic social observation [70]. In this study, only self-reported information on one neighborhood factor (i.e., neighborhood illicit drug selling) was available to us. Therefore, same-source bias (also known as shared-observer bias) might be an issue, as the exposure (perceived neighborhood illicit drug selling) and the outcomes (peer attitudes towards illicit drug and illicit drug use) were all assessed via self-report [71,72]. Because self-reported drug use information was collected, there might be some misclassification, in part due to social desirability bias. We recognize that we examined perception of one’s residential neighborhood, which is only one neighborhood context. Spatial polygamy asserts that people experience and interact with multiple neighborhood environments, which can influence their health and health behavior including drug use behaviors [73,74]. Because high school students spend significant amount of time at school [75], their school neighborhood environment may influence drug use behaviors. While we controlled for several confounding factors, residual confounding may also be a concern (e.g., we were unable to control for neighborhood poverty, residential stability and residential selection because these variables where not included in the survey). Finally, missing data (especially 14.3% missing for race/ethnicity and 23.0% missing for religiosity, with data missing not at random) is somewhat problematic. However, to maintain power and representativeness we included missing data indicators for covariates in all analyses.

## Conclusion

We found that perceived neighborhood drug selling was associated with lower peer disapproval of illicit drugs and more illicit drug use among a population-based nationally representative sample of U.S. high school seniors. Policy interventions to reduce “open” (visible) neighborhood drug selling (e.g., problem-oriented policing and modifications to the physical environment such as installing and monitoring surveillance cameras) may reduce illicit drug use and peer disapproval of illicit drugs.

## Competing interest

The authors report no competing interest. The authors alone are responsible for the content and writing of the paper.

## Authors’ contributions

DTD conceived the study, interpreted the results, and drafted the article. JJP assisted with the study design, performed the statistical analysis, and critically revised the manuscript for intellectual content. JHW interpreted the results and critically revised the manuscript for intellectual content. All authors have read and approved the final manuscript.
